# 4EBP1 senses extracellular glucose deprivation and initiates cell death signaling in lung cancer

**DOI:** 10.1038/s41419-022-05466-5

**Published:** 2022-12-27

**Authors:** Yanan Wang, Jiapeng Lei, Song Zhang, Xiaomei Wang, Jiangbo Jin, Yufeng Liu, Mingxi Gan, Yi Yuan, Longhua Sun, Xiaolei Li, Tianyu Han, Jian-Bin Wang

**Affiliations:** 1 Jiangxi Institute of Respiratory Disease, The First Affiliated Hospital of Nanchang University, Nanchang City, 330006 Jiangxi China; 2 Jiangxi Hospital of China-Japan Friendship Hospital, Nanchang City, 330052 Jiangxi China; 3 Jiangxi Clinical Research Center for Respiratory Diseases, Nanchang City, 330006 Jiangxi China; 4 School of Basic Medical Sciences, Nanchang Medical College, Nanchang City, 330006 Jiangxi China; 5 Department of Hepatobiliary and Pancreatic Surgery, The Second Affiliated Hospital, Zhejiang University School of Medicine, Hangzhou City, 310009 Zhejiang China; 6 Department of Pharmacy, Liaocheng People’s Hospital, Liaocheng City, 252000 Shandong China; 7 Department of Thoracic Surgery, The First Affifiliated Hospital of Nanchang University, Nanchang City, 330006 Jiangxi China; 8 School of Basic Medical Sciences, Nanchang University, Nanchang City, 330031 Jiangxi China; 9 Huankui Academy, Nanchang University, Nanchang City, 330031 Jiangxi China; 10 Departments of Pulmonary and Critical Care Medicine, The First Affiliated Hospital of Nanchang University, Nanchang City, 330006 Jiangxi China

**Keywords:** Non-small-cell lung cancer, Apoptosis, Phosphorylation, Stress signalling

## Abstract

Nutrient-limiting conditions are common during cancer development. The coordination of cellular glucose levels and cell survival is a fundamental question in cell biology and has not been completely understood. 4EBP1 is known as a translational repressor to regulate cell proliferation and survival by controlling translation initiation, however, whether 4EBP1 could participate in tumor survival by other mechanism except for translational repression function, especially under glucose starvation conditions remains unknown. Here, we found that protein levels of 4EBP1 was up-regulated in the central region of the tumor which always suffered nutrient deprivation compared with the peripheral region. We further discovered that 4EBP1 was dephosphorylated by PTPMT1 under glucose starvation conditions, which prevented 4EBP1 from being targeted for ubiquitin-mediated proteasomal degradation by HERC5. After that, 4EBP1 translocated to cytoplasm and interacted with STAT3 by competing with JAK and ERK, leading to the inactivation of STAT3 in the cytoplasm, resulting in apoptosis under glucose withdrawal conditions. Moreover, 4EBP1 knockdown increased the tumor volume and weight in xenograft models by inhibiting apoptosis in the central region of tumor. These findings highlight a novel mechanism for 4EBP1 as a new cellular glucose sensor in regulating cancer cell death under glucose deprivation conditions, which was different from its classical function as a translational repressor.

## Introduction

In order to acquire sufficient energy and metabolites to support rapid cell growth and proliferation, cancer cells are metabolically reprogrammed [1, 2]. Glucose is one of the key nutrients and cancer cells always absorb and metabolize glucose to a greater extent than normal cells [3]. During the rapid proliferation of solid tumors, the central region of the tumor always suffers deficient supplies of glucose due to insufficient angiogenesis [4]. Glucose starvation (GS) can trigger apoptosis, necrosis, autophagy or cell growth arrest in different cancer cell types depending on genetic, epigenetic and environmental clues [5–8]. However, how the cells response to these extracellular changes and the molecular mechanism of glucose deprivation-induced cell death has not been completely established.

Eukaryotic translation initiation factor 4E binding protein 1 (4EBP1) represses translation by interacting eIF4E and inhibiting eIF4E from recruiting 40S ribosomal subunits during translation [9–11]. In response to extracellular stimuli or stress conditions, 4EBP1 can be phosphorylated by kinases such as mTORC1, MAPK, AKT and the hyperphosphorylated 4EBP1 dissociates from eIF4E allowing for active cap-dependent translation [12–15]. In cancer cells, 4EBP1 regulates cell proliferation and survival by controlling translation initiation of oncogenic mRNAs [16–18]. Thus, understanding the molecular mechanism of 4EBP1 phosphorylation is urgent in order to fully explore the mystery of cancer cell growth. Until now, many studies have focused on discovering the kinases for 4EBP1 [19]. However, little is known about the dephosphorylation of 4EBP1. Besides, if there are any other mechanisms except for translational repression function for 4EBP1 in cancer progression is unknown.

In this study, we found for the first time that 4EBP1 could regulate cancer cell death under glucose deprivation conditions. Glucose starvation (GS) induced the upregulation of total 4EBP1 protein accompanied with a shift from the highly phosphorylated state to less phosphorylated state in NSCLC (non-small cell lung cancer) cells. PTPMT1 (protein tyrosine phosphatase localized to the Mitochondrion 1) dephosphorylated and stabilized 4EBP1 in this process. We also identified HERC5 (HECT and RLD domain containing E3 ubiquitin protein ligase 5) as the E3 ligase targeting hyperphosphorylated 4EBP1 for degradation. Furthermore, 4EBP1 interacted with STAT3 (signal transducer and activator of transcription-3) to prevent JAK (Janus-activated kinases) or ERK (extracellular signal-regulated kinase) from phosphorylation and activation of STAT3, leading to decreased expression of anti-apoptotic genes and resulting in apoptosis. Taken together, these findings elucidated a key role for 4EBP1 to regulate cell viability in response to glucose starvation conditions, which differed from its translational repression function.

## Results

### Glucose deprivation induces dephosphorylation and stabilization of 4EBP1 in NSCLC cells

**Fig. 1:**
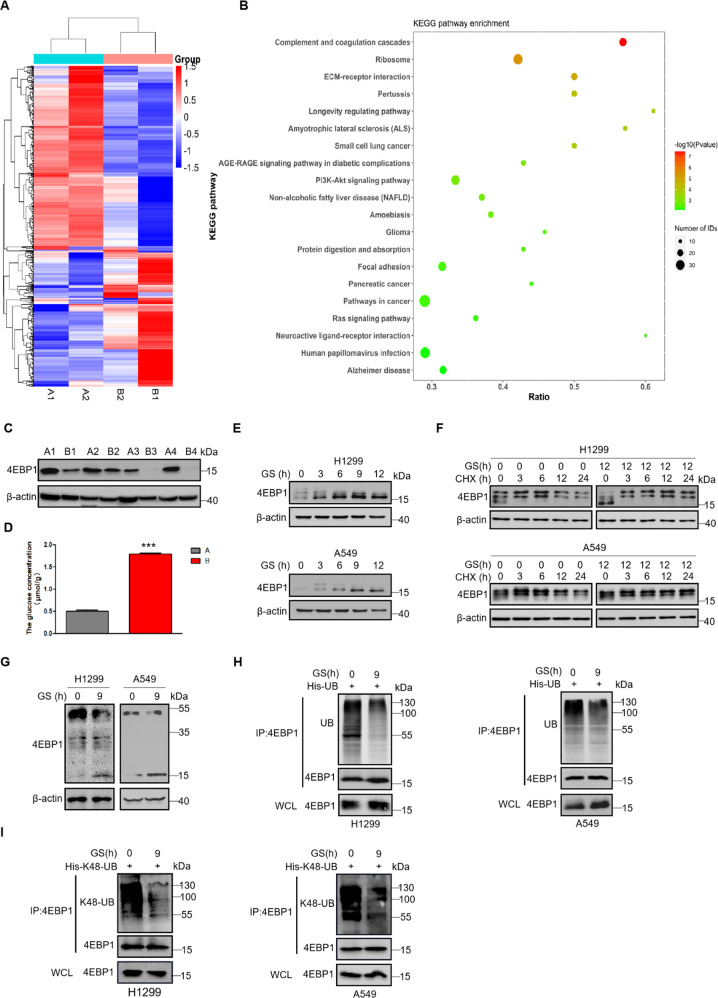
Glucose deprivation induces dephosphorylation and stabilization of 4EBP1 in NSCLC cells. **A**, **B** Proteomic analysis. The samples using the central region (**A**) and peripheral region (**B**) of the tumors derived from two lung adenocarcinoma patients. Cluster heat map of 652 differentially expressed proteins between the central region and peripheral region of the tumors (**A**). KEGG enrichment bubble diagram: according to the results of KEGG annotation and offificial classifification, the differential proteins were functionally classified and conducted enrichment analysis (**B**). **C** Western blot analysis of 4EBP1 levels in the paired, tumor-central region (**A**) and tumor-peripheral region (**B**) derived from four lung adenocarcinoma patients. **D** The glucose concentration was detected using the central region (**A**) and peripheral region (**B**) of the tumors derived from the lung adenocarcinoma patient. **E** Western blot analysis of 4EBP1 expression in H1299 and A549 cells under normal (0 h) or glucose starvation (GS) conditions for 3, 6, 9 and 12 h. **F** H1299 and A549 cells were treated with CHX at 50 μg/ml for 0, 3, 6, 12, 24 h under normal or glucose starvation conditions (12 h). Western blot analysis of 4EBP1 expression. **G** Western blot analysis of 4EBP1 phosphorylation levels on Phos-Tag gels in H1299 and A549 cells under normal or glucose starvation conditions for 9 h. **H** H1299 and A549 cells transfected with plasmid His-UB were treated with normal or glucose starvation (9 h). The immunoprecipitation was done using anti-4EBP1 antibody followed by western blot. WCL: the whole cell lysate. **I** H1299 and A549 cells transfected with plasmid His-K48-UB were treated with normal or glucose starvation (9 h). The immunoprecipitation was done using anti-4EBP1 antibody followed by western blot.

To explore the molecular mechanisms for tumor cell death in the central region, we separated the central region and peripheral region of the tumors derived from two lung adenocarcinoma patients, and performed proteomic analysis. The results showed that 377 proteins were up-regulated and 275 proteins were down-regulated in the central region compared with the peripheral region of the tumor (Fig. 1A and Supplementary Table 1). We analyzed the up-regulated proteins using KEGG pathway analysis, and found that the longevity regulating pathway had the highest rich ratios in the top 20 signal pathways with significant changes (Fig. 1B and Supplementary Table 2). Interestingly, 4EBP1 gene changed most in this pathway. The protein expression of 4EBP1 in the peripheral region of the tumor was almost undetectable, while 4EBP1 showed a relatively high expression in the central region (Supplementary Table 1). Using western blot, we discovered the same phenomenon that 4EBP1 showed higher expression in the central region compared with the peripheral region of the tumors derived from lung adenocarcinoma patients (Fig. 1C). Thus, we suspected that 4EBP1 might play a key role in cell death progression in the central region of the tumor. Glucose is essential for tumor cell survival and tumor initiation and progression, the central region of tumor always have an insufficient supply of glucose due to inadequate angiogenesis [4]. We confirmed this by assessing the glucose levels in the central region and peripheral region of the tumors. Figure 1D showed that the glucose levels in the central region was much lower than that in peripheral region. According to these phenomenons, we had great interests to investigate if 4EBP1 could respond to glucose starvation (GS) in NSCLC. According to differentially phosphorylated forms [20–23], the hyperphosphorylated 4EBP1 migrates more slowly than the hypophosphorylated forms. 4EBP1 had a shift from the highly phosphorylated 4EBP1 toward the less phosphorylated forms under glucose starvation (Fig. 1E). However, the mRNA levels of 4EBP1 had no significant changes under the same conditions (Supplementary Fig. 1A), indicating that glucose starvation might affect the protein phosphorylation and stability of 4EBP1. We tested the stability of 4EBP1 protein in NSCLC cells under normal and GS conditions. Compared to the normal conditions, GS attenuated the degradation rate of 4EBP1. Furthermore, the transition rate from hypophosphorylated 4EBP1 to hyperphosphorylated 4EBP1 was slowed down under GS (Fig. 1F). To further demonstrate glucose deprivation affected the phosphorylation of 4EBP1, we detected the phosphorylation levels of 4EBP1 on Phos-Tag gels. Figure 1G showed that the phosphorylation of 4EBP1 decreased under GS. Ubiquitin-mediated protein degradation is closely associated with protein stability. We further tested the ubiquitination of 4EBP1 under GS. As shown in Fig. 1H, GS decreased the ubiquitination of 4EBP1 in NSCLC cells. K48-polyubiquitin chains are the major signal for proteasome-mediated degradation [24, 25], and GS significantly reduced the K48-linked ubiquitination of 4EBP1 in NSCLC cells (Fig. 1I). K63-polyubiquitin chains are the major signal for lysosome-mediated degradation [26, 27], but we found the K63-linked ubiquitination of 4EBP1 had no changes under GS (Supplementary Fig. 1B). Therefore, these intriguing results suggested that GS reduced the phosphorylation levels of 4EBP1 and stabilized 4EBP1 via inhibiting its K48-linked ubiquitination.

### PTPMT1 dephosphorylates and stabilizes 4EBP1 under glucose deprivation conditions

**Fig. 2:**
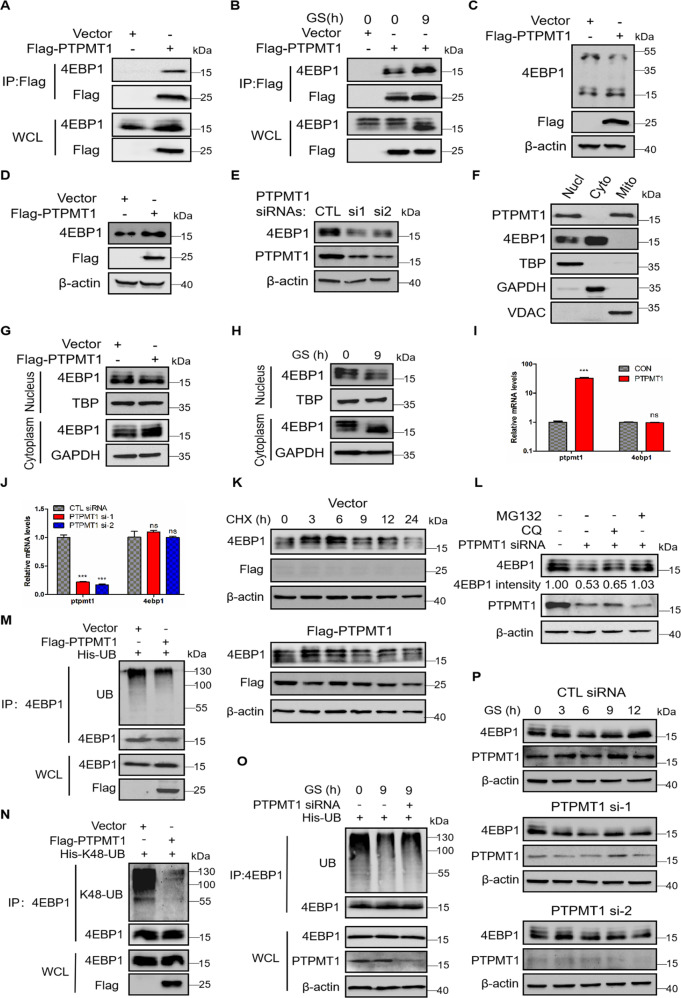
PTPMT1 dephosphorylates and stabilizes 4EBP1 under glucose deprivation conditions. **A**, **B** A549 cells were transfected with plasmid Flag-PTPMT1 or vector under normal (**A**) or glucose starvation conditions for 9 h (**B**). The immunoprecipitation was performed followed by western blot. WCL: the whole cell lysate. **C** A549 cells transfected with plasmid Flag-PTPMT1 or vector were analyzed using Phos-Tag gels, followed by western blot. **D**, **E** A549 cells were transfected with plasmid Flag-PTPMT1 (**D**) or PTPMT1 siRNAs (**E**). Western blot analysis of 4EBP1 expression. **F** The nuclear, cytoplasmic and mitichondrial fractions were separated in A549 cells. Then western blot was done. Nucl: nucleus, Cyto: cytoplasm, Myto:mitichondria. **G**, **H** A549 cells were transfected with plasmid Flag-PTPMT1 (**G**) or treated with glucose starvation for 9 h (**H**). Western blot analysis of 4EBP1 levels in the nuclear and cytoplasmic fractions. **I**, **J** A549 cells were transfected with plasmid Flag-PTPMT1 (**I**) or PTPMT1 siRNAs (**J**). q-PCR analysis of 4EBP1 expression. The data represent the averages of three independent experiments (mean ± SD). ns: *P* > 0.05, ***P < 0.001. **K** A549 cells transfected with plasmid Flag-PTPMT1 or vector were treated with CHX (50 μg/ml) for different times. Western blot analysis of 4EBP1 expression. **L** A549 cells transfected with either control siRNA or PTPMT1 siRNAs were treated with chloroquine (CQ, 20 μM) or MG132 (20 μM). Western blot analysis of 4EBP1 expression. **M**, **N** A549 cells were transfected with plasmids Flag-PTPMT1 and His-UB (**M**) or His-K48-UB (**N**). Immunoprecipitation assay was performed and the ubiquitination (**M**) or K48-linked ubiquitination (**N**) of 4EBP1 was checked by western blot. **O** A549 cells transfected with plasmid His-UB and control siRNA or PTPMT1 siRNAs were treated with normal (0 h) or glucose starvation for 9 h. Immunoprecipitation assay was performed and the ubiquitination of 4EBP1 was detected by western blot. **P** A549 cells transfected with either control siRNA or PTPMT1 siRNAs were treated with normal or glucose starvation for different times. Western blot analysis of 4EBP1 expression.

To further elucidate the regulatory mechanism of 4EBP1 under GS, we tried to figure out the phosphatase of 4EBP1 by mass spectrometric analysis. Among the identified proteins, PTPMT1 caught our attention. Protein levels of PTPMT1 had no significant changes under glucose deprivation conditions (Fig. S2A, B). But co-immunoprecipitation experiment showed that PTPMT1 interacted with 4EBP1 (Fig. 2A) and this interaction was enhanced under GS (Fig. 2B). We further checked the phosphorylation levels of 4EBP1 on Phos-Tag gels. Figure 2C showed that overexpressing PTPMT1 decreased the phosphorylation of 4EBP1, indicating that PTPMT1 was the phosphatase for 4EBP1. We next detected the effects of PTPMT1 on 4EBP1 expression. Overexpressing PTPMT1 up-regulated the protein levels of 4EBP1 (Fig. 2D) while knocking down PTPMT1 reduced 4EBP1 protein (Fig. 2E). However, a confusing question is that PTPMT1 is known to reside in the mitochondria [28], it was still unclear if PTPMT1 located in other organelle, while 4EBP1 is demonstrated to localize in cytoplasm and nucleus [19]. In an effort to profile the colocalization possibility of PTPMT1 and 4EBP1, we detected the location of PTPMT1 and 4EBP1 in A549 cells. To our surprise, PTPMT1 was located not only in mitochondria but also in nucleus, and 4EBP1 resided in the nucleus and cytoplasm (Fig. 2F). Next we checked the protein levels of 4EBP1 in the nucleus and cytoplasm in A549 cells when overexpressing PTPMT1. Interestingly, the protein levels of total 4EBP1 and hypophosphorylated 4EBP1 were significantly increased in the cytoplasm but only a bit reduction in the nucleus (Fig. 2G). Similar results were also obtained in cells treated with glucose starvation (Fig. 2H). These results revealed that PTPMT1 interacted with 4EBP1 and then dephosphorylated 4EBP1 in the nucleus, leading to the translocation of 4EBP1 into the cytoplasm. We also found that PTPMT1 did not affect the mRNA levels of 4EBP1 (Fig. 2I, J). However, overexpressing PTPMT1 attenuated the degradation rate of 4EBP1 (Fig. 2K). Consistently, the degradation rate of 4EBP1 was accelerated when PTPMT1 knockdown (Fig. S2C). The decreased protein levels of 4EBP1 by PTPMT1 knockdown could be recovered by adding MG132, a proteasomal inhibitor, but not the lysosomal inhibitor chloroquine (CQ) (Fig. 2L), indicating a proteasome-mediated degradation. As shown in Fig. 2M, the ubiquitination levels of 4EBP1 were reduced when overexpressing PTPMT1, and Fig. 2N showed that overexpressing PTPMT1 decreased the K48-linked ubiquitination of 4EBP1. To figure out the exact molecular mechanisms of PTPMT1 in regulating 4EBP1 protein levels under GS conditions, we detected the ubiquitination and protein levels of 4EBP1 with PTPMT1 knockdown under GS. The results indicated that PTPMT1 knockdown could recover the decreased ubiquitination levels of 4EBP1 (Fig. 2O) and attenuated the GS-induced upregulation of 4EBP1 protein (Fig. 2P). All the results demonstrated that PTPMT1 dephosphorylated and stabilized 4EBP1 by preventing 4EBP1 from being targeted for ubiquitin-mediated proteasomal degradation under glucose starvation conditions.

### Glucose deprivation suppresses HERC5-mediated ubiquitination and degradation of 4EBP1

**Fig. 3:**
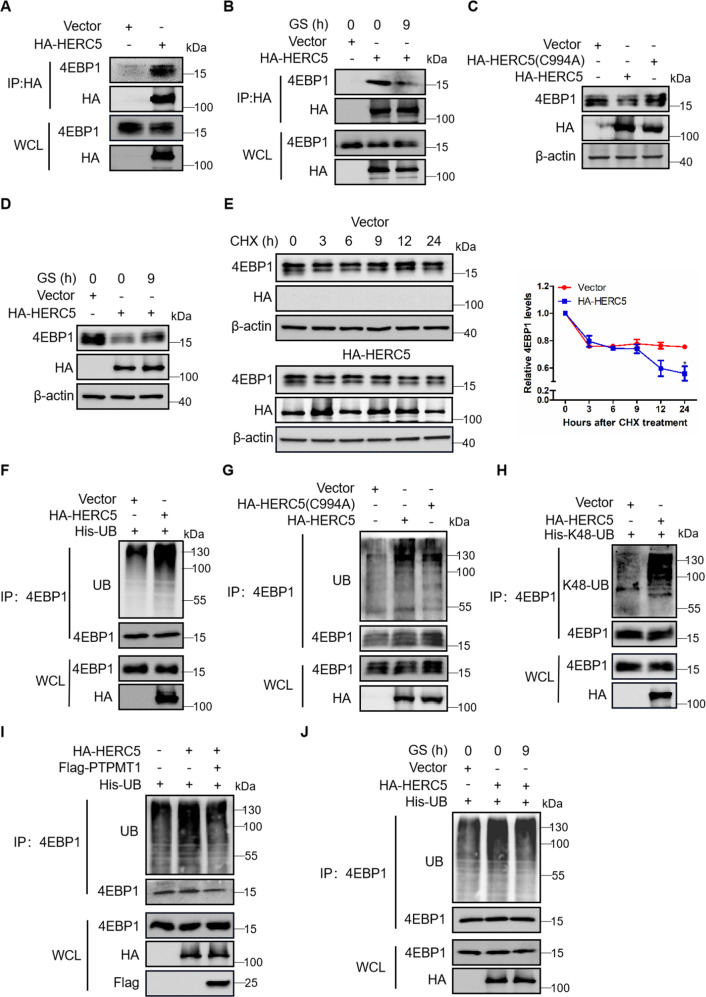
Glucose deprivation suppresses HERC5-mediated ubiquitination and degradation of 4EBP1. **A**, **B** A549 cells were transfected with plasmid HA-HERC5 or vector under normal (**A**) or glucose starvation conditions for 9 h (**B**). The immunoprecipitates using anti-HA antibody were blotted with related antibodies. WCL: the whole cell lysate. **C** A549 cells were transfected with plasmid HA-HERC5 or mutant plasmid HA-HERC5 (C994A). Western blot analysis of 4EBP1 expression. **D** A549 cells transfected with plasmid HA-HERC5 or vector were treated with normal (0 h) or glucose starvation for 9 h. Western blot analysis of 4EBP1 expression. **E** A549 cells transfected with plasmid HA-HERC5 or vector were treated with CHX at 50 μg/ml for 0, 3, 6, 9, 12 and 24 h. Western blot (left panel) and statistical analysis (right panel) of 4EBP1 expression, *P < 0.05. **F** A549 cells were transfected with plasmids His-UB and HA-HERC5 or vector. The immunoprecipitates using anti-4EBP1 antibody were blotted with anti-UB antibody. **G** A549 cells were transfected with plasmids HA-HERC5 or HA-HERC5 (C994A). The immunoprecipitates using anti-4EBP1 antibody were blotted with anti-UB antibody. **H** A549 cells were transfected with plasmids His-K48-UB and HA-HERC5 or vector. The immunoprecipitates using anti-4EBP1 antibody were blotted with the K48-linkage-specific polyubiquitin antibody. **I** A549 cells were transfected with plasmids His-UB and HA-HERC5 or Flag-PTPMT1. The immunoprecipitates using anti-4EBP1 antibody were blotted with anti-UB antibody. **J** A549 cells transfected with plasmids His-UB and HA-HERC5 or vector were treated with normal or glucose starvation for 9 h. The immunoprecipitates using anti-4EBP1 antibody were blotted with anti-UB antibody.

In the mass spectrometric analysis, we also found another possible binding partner of 4EBP1, the HERC5 which is a E3 ubiquitin ligase and resides in the cytoplasm [29, 30]. We found that protein levels of HERC5 were down-regulated in GS-treated cells (Fig. S3A) and the central region of tumor (Fig. S3B), this trend inversely correlated with the expression of 4EBP1. Furthermore, co-immunoprecipitation experiment proved that HERC5 interacted with 4EBP1 (Fig. 3A) and the interaction between HERC5 and 4EBP1 was attenuated by GS (Fig. 3B). We found that overexpression of HERC5 reduced the protein levels of 4EBP1 (Fig. S3C) but had no effects on the mRNA levels of 4EBP1 (Fig. S3D). Using the dominant negative mutant HERC5-C994A, we found that overexpressing HERC5-C994A could not decrease the protein levels of 4EBP1 compared with HERC5 wild-type (Fig. 3C). Interestingly, the decreased protein levels of 4EBP1 by overexpressing HERC5 were rescued by GS (Fig. 3D). So HERC5 might be the E3 ligase for 4EBP1. Figure 3E showed that the degradation rate of 4EBP1 was accelerated when HERC5 overexpressed and the ubiquitination levels of 4EBP1 were increased when overexpressing HERC5 but not HERC5-C994A (Fig. 3F, G). Furthermore, overexpressing HERC5 elevated the K48-linked ubiquitination of 4EBP1 (Fig. 3H), and this increased ubiquitination of 4EBP1 could be attenuated by overexpressing PTPMT1 (Fig. 3I). Similar results were also obtained in cells treated with GS (Fig. 3J). These results proved that glucose starvation promoted 4EBP1 dephosphorylation and inhibited HERC5-mediated ubiquitination and degradation of 4EBP1.

### 4EBP1 plays a crucial role in cell apoptosis under glucose deprivation conditions

**Fig. 4:**
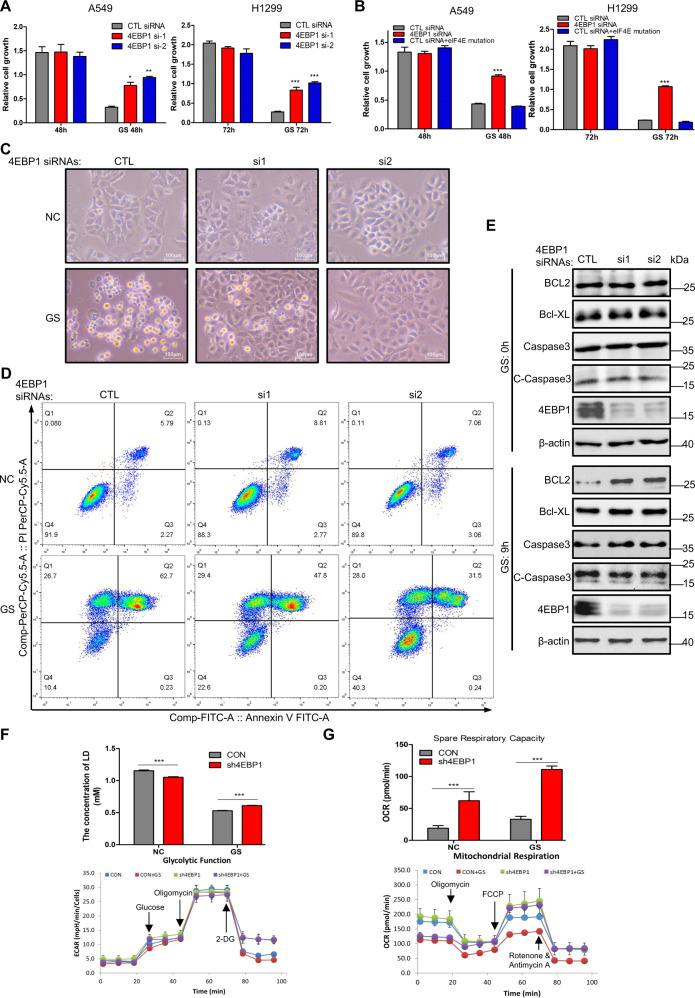
4EBP1 plays a crucial role in cell apoptosis under glucose deprivation conditions. **A**, **B** A549 and H1299 cells transfected with either control (CTL) siRNA or 4EBP1 siRNAs (**A**), A549 and H1299 cells were transfected with CTL siRNAs or 4EBP1 siRNA or cotransfected with CTL siRNAs and mutant plasmid eIF4E-S209E (**B**). Cells were treated with normal or glucose starvation. Cell growth rate was assessed by MTT assay. The data represent the averages of three independent experiments (mean ± SD). *P < 0.05, **P < 0.01, ***P < 0.001. **C** A549 cells transfected with either control siRNA or 4EBP1 siRNAs were treated with normal or glucose starvation for 16 h. The changes of cell morphological was investigated (scale bar = 100 µm, magnification: ×100). **D** A549 cells transfected with either control siRNA or 4EBP1 siRNAs were treated with normal or glucose starvation for 16 h. Cell apoptosis analysis was done by flow cytometry. **E** A549 cells transfected with either control siRNA or 4EBP1 siRNAs were treated with normal (0 h) or glucose starvation. The expression levels of the apoptosis related proteins were checked by western blot. C-Caspase3: Cleaved-Caspase3. **F**, **G** parental A549 (CON) and A549 stable cell lines with 4EBP1 knockdown (A549-sh4EBP1) were treated with normal or glucose starvation for 12 h. Lactic acid levels were monitored (**F**, upper panel). ECAR (**F**, lower panel) and OCR (**G**) measurements were done.

We next want to elucidate the biological function of 4EBP1 under GS conditions. Figure 4A showed that 4EBP1 knockdown only slightly retarded cell growth in normal conditions, however, cell death induced by glucose starvation is rescued by 4EBP1 knockdown. Phosphorylation of 4EBP1 releases eIF4E, and then eIF4E is further activated by phosphorylation at S209 [31, 32]. To determine if the effect of 4EBP1 on apoptosis under GS was dependent on its translation suppressor function, we overexpressed the constitutive activated mutant eIF4E-S209E in NSCLC cells. We found that overexpressing eIF4E-S209E could not rescue cells from apoptosis under GS (Fig. 4B). We further checked protein levels of p-eIF4E-S209 under GS. These results showed that protein levels of p-eIF4E-S209 were up-regulated but not down-regulated in GS-treated cells (Fig. S4A) and the central region of tumor (Fig. S4B). These data indicated that 4EBP1 affected cell apoptosis under GS was independent of its translation suppressor function. Furthermore, there were obvious morphological changes under GS when 4EBP1 was knocked down. Glucose-starved cells transfected with control siRNA were rounded, shrunken in size, and the significant cell death was noted. 4EBP1 knockdown had no significant effects on cell morphology in normal glucose conditions. However, the cells with 4EBP1 knockdown displayed efficient rescue of the abnormal cell morphology induced by GS (Figs. 4C and S4C). Annexin V-FITC and propidium iodide based FACS analysis of cell apoptosis demonstrated that glucose-starved A549 cells showed nearly 63% cell apoptosis, whereas cells with 4EBP1 knockdown showed apoptosis about 48 and 32% (Fig. 4D). In addition, 4EBP1 knockdown had no notably effects on apoptosis in normal conditions (Fig. 4D). Similar results were also obtained in H1299 cells (Fig. S4D). We further demonstrated that the expression of anti-apoptotic factors (Bcl-XL, BCL2) were increased and apoptosis marker Caspase-3 were less cleaved in A549 cells with 4EBP1 knockdown in glucose starvation conditions not in normal conditions (Fig. 4E). We further assessed glycolytic function by measurement of lactic acid levels and the extracellular acidifcation rate (ECAR). We found that 4EBP1 knockdown reduced lactic acid levels in normal conditions but increased lactic acid levels in glucose starvation conditions compared with control group (Fig. 4F). Consistent with the increase of lactic acid levels when 4EBP1 was knocked down under GS, we observed a signifcant increase of ECAR in cells with 4EBP1 knockdown when HK2 inhibitor 2-DG was added to inhibit glycolysis (Fig. 4F). This indicated that 4EBP1 knockdown might mobilize other metabolic pathways to supplement glycolysis under glucose starvation or glycolysis inhibition. We next assessed mitochondrial activity by measurement of the oxygen consumption rate (OCR). Spare respiratory capacity was used as an indicator of cellular fitness to cell stress. Results indicated that 4EBP1 knockdown increased the spare respiratory capacity of mitochondrial especially in GS conditions (Fig. 4G), suggesting cells with 4EBP1 knockdown had a greater cellular adaptability to GS conditions. These results suggested that 4EBP1 knockdown inhibited GS-triggered apoptotic but had no significant effects in normal conditions.

### 4EBP1 induces apoptosis via inactivation of STAT3 under glucose deprivation conditions

**Fig. 5:**
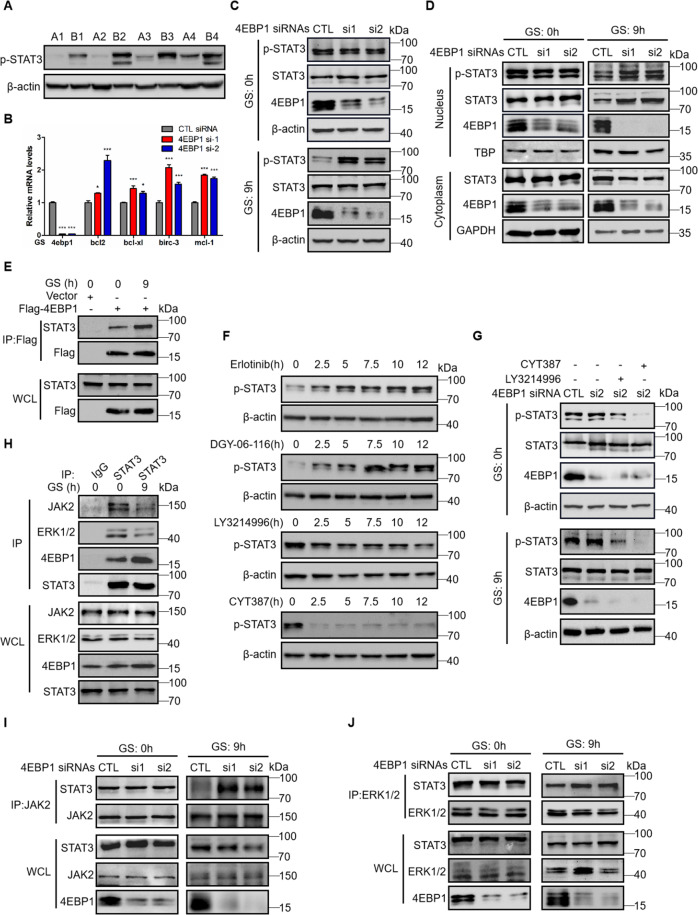
4EBP1 induces apoptosis via inactivation of STAT3 under glucose deprivation conditions. **A** The protein levels of p-STAT3 (Y705) were checked by western blot using the paired, tumor-central region (**A**) and tumor-peripheral region (**B**) derived from four lung adenocarcinoma patients. **B–D** A549 cells transfected with either control (CTL) siRNA or 4EBP1 siRNAs were treated with normal (0 h) or glucose starvation for 9 h. **B** q-PCR analysis of STAT3 downstream target genes expression. The data represent the averages of three independent experiments (mean ± SD). *P < 0.05, ***P < 0.001. **C** Western blot analysis of p-STAT3 (Y705) expression. **D** Western blot analysis of STAT3 and p-STAT3 (Y705) expression in the nuclear and cytoplasmic fractions. **E** A549 cells transfected with plasmid Flag-4EBP1 or vector were treated with normal or glucose starvation for 9 h. The immunoprecipitation was done using anti-Flag antibody followed by western blot. WCL: the whole cell lysate. **F** A549 cells were treated with Erlotinib, DGY-06-116, LY3214996 or CYT387. Western blot analysis of p-STAT3 (Y705) expression. **G** A549 cells transfected with either control siRNA or 4EBP1 siRNAs were treated with CYT387 or LY3214996 for 10 h in normal or glucose starvation conditions. Western blot analysis of p-STAT3 (Y705) expression. **H** A549 cells were treated with normal or glucose starvation for 9 h. The immunoprecipitation was done using anti-STAT3 antibody followed by western blot. **I**,**J** A549 cells transfected with either control siRNA or 4EBP1 siRNAs were treated with normal or glucose starvation. The immunoprecipitates using anti-JAK2 (**I**) or anti-ERK1/2 (**J**) antibody were blotted with related antibodies.

STAT3 is an important transcription factor that regulates a series of anti-apoptotic genes to mediate cell survival (bcl-xl, bcl2, survivin, mcl-1 et al.) [33–37]. Phosphorylation of STAT3 at Y705 leads to its dimerization, nuclear translocation, DNA binding and transcription initiation [38, 39]. Thus, we assessed the expression of p-STAT3-Y705 in the central region and peripheral region of lung cancer. We found that the protein levels of p-STAT3 in the central region of tumor were lower compared to those in the peripheral region of tumor (Fig. 5A). It was completely opposite with the protein levels of 4EBP1 (Fig. 1C), indicating that 4EBP1 might associate with STAT3. Then q-PCR analysis showed that the mRNA levels of STAT3 target genes including bcl2, mcl-1, survivin and bcl-xl were increased under GS when 4EBP1 was knocked down (Fig. 5B), indicating that 4EBP1 suppressed the activation of STAT3 under GS conditions. We further assessed the expression of p-STAT3-Y705 in A549 cells when 4EBP1 was knocked down (Fig. 5C). The results showed that knocking down 4EBP1 increased the phosphorylation levels of STAT3 in GS conditions not in normal conditions. Interestingly, the expression of p-STAT3-Y705 and STAT3 were elevated in the nucleus, while, protein levels of STAT3 were deceased in cytoplasm in GS conditions not in normal conditions when 4EBP1 was knocked down (Fig. 5D). These data indicated that knocking down 4EBP1 induced the phosphorylation and translocation of STAT3 from cytoplasm to nucleus under GS and promoted the expression of anti-apoptotic genes. We further investigated the interaction between 4EBP1 and STAT3. Co-immunoprecipitation experiment showed that 4EBP1 interacted with STAT3 and this interaction was increased by GS (Fig. 5E). Previous study reported that JAK, ERK, EGFR kinase and Src could phosphorylate STAT3 on Y705 and activate STAT3 [40–43]. To verify this, we treated A549 cells with EGFR kinase inhibitor Erlotinib, Src inhibitor DGY-06-116, ERK inhibitor LY3214996 and JAK1/2 inhibitor CYT387 to detect the expression of p-STAT3 (Fig. 5F). We found only CYT387 and LY3214996 could inhibit the phosphorylation of STAT3. Furthermore, the increased protein levels of p-STAT3 by knocking down 4EBP1 under GS were attenuated when treating with CYT387 or LY3214996 in A549 cells (Fig. 5G). Although protein levels of p-STAT3 had no effects by knocking down 4EBP1 under normal conditions, they were also attenuated by treating with CYT387 or LY3214996 (Fig. 5G). These data indicated that JAK1/2 or ERK could phosphorylate STAT3 in A549 cells both in normal and glucose starvation conditions. We assessed the interaction between JAK2 or ERK and STAT3 under GS. As shown in Fig. 5H, the interaction between JAK2 or ERK and STAT3 was attenuated, while the interaction between 4EBP1 and STAT3 was enhanced under GS. Further, the interactions between JAK2 or ERK and STAT3 were increased when knocking down 4EBP1 in GS conditions not in normal conditions (Fig. 5I, J). These results indicated that activation of STAT3 by JAK2 or ERK was elevated when 4EBP1 knockdown under GS. Collectively, these data revealed that 4EBP1 interacted with STAT3 to prevent JAK2 or ERK from phosphorylating and activating STAT3, resulting in the apoptosis under GS.

### 4EBP1 inhibits tumor progression under glucose deprivation conditions in vivo

**Fig. 6:**
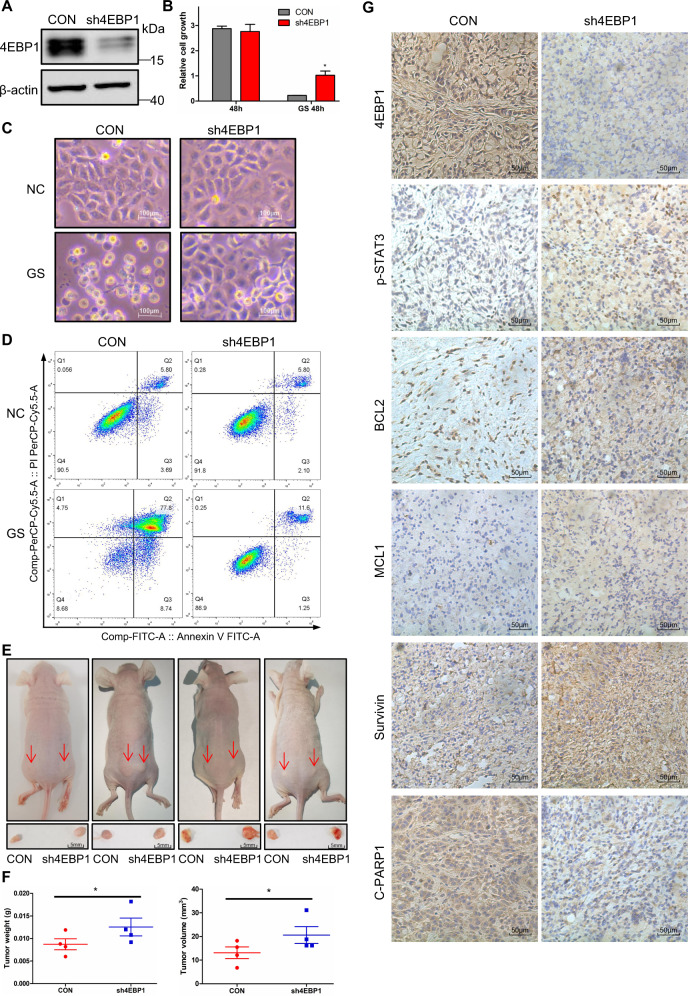
4EBP1 inhibits tumor progression under glucose deprivation conditions in vivo. **A** Western blot analysis of 4EBP1 expression in parental A549 and A549-sh4EBP1 cells. **B** Parental A549 and A549-sh4EBP1 cells were treated with normal or glucose starvation for 48 h. Cell growth rate was assessed by MTT assay. The data represent the averages of three independent experiments (mean ± SD). *P < 0.05. **C** Parental A549 and A549-sh4EBP1 cells were treated with normal or glucose starvation for 16 h. The changes of cell morphological was investigated (scale bar = 100 µm, magnification: ×100). **D** Parental A549 and A549-sh4EBP1 cells were treated with normal or glucose starvation for 20 h. Cell apoptosis analysis was done by flow cytometry. **E**, **F** In vivo xenograft assay was performed using parental A549 and A549-sh4EBP1 cells, tumors were photographed (**E**), and their weights and volumes were measured (**F**). The *P* value was calculated by paired *t*-test. *P < 0.05. **G** Immunohistochemical staining in the tumor-central region of tumor induced by parental A549 and A549-sh4EBP1 cells for 4EBP1, p-STAT3, BCL2, MCL1, Survivin and Cleaved PARP1 (C-PARP1). Scale bars: 50 μm, magnification: ×200.

To elucidate the effect of 4EBP1 on tumor progression, we generated A549 stable cell lines with 4EBP1 knockdown (A549-sh4EBP1) and performed xenograft experiment. The knockdown efficiency of A549-sh4EBP1 were assessed by western blot (Fig. 6A). MTT assay showed that cell death induced by glucose starvation is rescued in A549-sh4EBP1 cells (Fig. 6B). Under glucose starvation, parental A549 cells were rounded, shrunken in size, and significant cell death was noted, but A549-sh4EBP1 cells displayed efficient rescue with normal morphology under the same conditions (Fig. 6C). FACS analysis showed that the number of apoptotic cells was greatly reduced in A549-sh4EBP1 cells than parental A549 cells under GS conditions (Fig. 6D). The xenografts of A549-sh4EBP1 cells displayed a increased tumor size and weight compared with parental A549 cells (Fig. 6E, F). To confirm our results, we detected the expression of apoptosis markers by immunohistochemistry in the central region of the tumor which was deficient in glucose supply. Compared with the central region in xenografts of parental A549 cells, p-STAT3-Y705 and its target anti-apoptotic genes were significantly increased in the central region in xenografts of A549-sh4EBP1 cells. The cleaved PARP1 was dramatically reduced in xenografts of A549-sh4EBP1 cells compared with parental A549 cells (Fig. 6G), suggesting less apoptosis in 4EBP1-knockdown xenografts. Similar results were also obtained in the central region and peripheral region in xenografts of parental A549 cells (Fig. S5A). These results further indicated that 4EBP1 inhibited tumor progression under glucose deprivation.

## Discussion

Since nutrient-limiting conditions are universal during cancer progress [44], how cancer cells integrate the starvation signaling and respond to these changes are a fundamental question in cell biology and has not been fully understood. This study provided a detailed molecular mechanism on how cells sensed glucose starvation and responded to these changes through regulating the phosphorylation and stabilization of 4EBP1. Our model indicated that 4EBP1 was a new cellular glucose sensor. Under glucose deprivation, 4EBP1 played a vital role to suppress STAT3 signaling in response to glucose starvation in NSCLC cells and initiated the cell death signaling.

4EBP1 has been reported to possess dual roles in tumor progression. For one thing, 4EBP1 has the tumor suppressive activity where 4EBP1 inhibits eIF4E and, thus, blocks mRNA translation and proliferation [45, 46]. This is confirmed by increased levels of phosphorylated and hence inactive 4EBP1 in various human cancer [19]. For another, 4EBP1 has the tumorigenic roles by promoting tumor adaptation to metabolic and genotoxic stress by selectively blocking or enhancing the translation of related transcripts [47]. In our study, we found that 4EBP1 inhibition by siRNAs had no obvious effects on cell apoptosis under normal conditions but alleviated cell apoptosis under glucose starvation conditions, suggesting that the contribution of 4EBP1 to tumor progression could differ depending on the genetic, epigenetic and environmental clues.

Previous studies have demonstrated that ~30% of 4EBP1 resides in the nucleus where 4EBP1 retains eIF4E in the nucleus to regulate the availability of eIF4E for the cytoplasmic translational machinery [48]. However, the function of the remaining 70% 4EBP1 in the cytoplasm remains unknown until now. Previous study showed that 4EBP1 interacted directly with PPARγ to regulate lipid synthesis in bovine mammary epithelial cells [49]. This suggested a possible role of 4EBP1 independent of the translation-repressor function. Our study for the first time, elucidated that 4EBP1 played an important physiological role to trigger apoptosis in the cytoplasm in response to glucose deprivation in NSCLC, which was different from the translation-repressor function. Dephosphorylated 4EBP1 translocated into the cytoplasm from nucleus and prevented the activation of STAT3, which then induced apoptosis.

PTPMT1 was reported to reside only in the mitochondria [28] and has an obvious preference for lipid substrates rather than protein substrates [50]. But Nath et al. showed that tyrosine phosphorylated SDHA was a substrate of PTPMT1 in zebrafish [51]. Until now, little is known about the protein substrate of PTPMT1. Here, we found that PTPMT1 was located not only in mitochondria but also in nucleus and had phosphatase activity to dephosphorylate 4EBP1 protein. Dephosphorylation of 4EBP1 by PTPMT1 stabilized 4EBP1 to prevent HERC5-mediated degradation in ubiqutin-proteasome pathway. Although early report proposed that protein phosphatase 2 A maybe associate with 4EBP1, which likely dephosphorylated 4EBP1 [52], the interaction and dephosphorylation of 4EBP1 by phosphatase 2 A were uncertain. Thus, this is the first study to identify the phosphatase for 4EBP1. 4EBP1 can be phosphorylated in a hierarchical manner at multiple, disparate sites and it needs numerous kinases [14, 53]. Activation of a phosphatase may allow fast dephosphorylation of these different sites without suppressing all the kinases responsible for their phosphorylation. So targeting phosphatase for cancer treatment may have more advantages over targeting kinase. Thus, our study provides a new therapeutic strategy toward cancer treatment that activating PTPMT1 to dephosphorylate 4EBP1 which leads to apoptosis from the core region of the tumor.

## Materials and methods

### Antibodies and reagents

The primary antibodies used are as follows: UB, PTPMT1, Caspase-3, STAT3, TBP, ERK1/2, JAK2, VDAC, β-actin, Flag, GAPDH (Proteintech, 10201-2-AP, 11493-1-AP, 19677-1-AP, 10253-2-AP, 22006-1-AP, 16443-1-AP, 17670-1-AP, 10866-1-AP, 66009-1-Ig, 66008-4-Ig, 60004-1-Ig), K48-linkage-specific polyubiquitin, 4EBP1, BCL2, Bcl-Xl, K63-linkage-specific polyubiquitin, P-STAT3-Tyr705 (Cell Signaling Technology, 8081, 9644, 4223, 2764, 5621, 9145), HA (Invitrogen, 26183). The reagents used are as follows: MG132 (Biovision, 1791-5), Chloroquine, DMSO and Cycloheximide (CHX) (Sigma, RNBH9960, C6628, C7698), MG132, MTT, Erlotinib, DGY-06-116, LY3214996, CYT387, PMSF (MedChemExpress, HY13259, HY-15924, HY-50896, HY-136605, HY-101494, HY-10961, HY-B0496). The plasmids used are as follows: pEnCMV-HA-HERC5 (MiaoLing Plasmid Sharing Platform), pCMV6-Entry-Myc-DDK-4EBP1, pCMV6-Entry-Myc-DDK-PTPMT1, (OriGene, RC201348, RC215376). The siRNAs that target 4EBP1 (HSS141934, HSS141935), PTPMT1 (HSS174384, HSS174385) and Stealth RNAi^TM^ siRNA Negative Control (12935300) were purchased from Invitrogen. The plasmids or siRNAs were transfected with the transfection reagent kit (OriGene, TT210003, TT320001).

### Patient samples

Samples of human lung adenocarcinoma were the gift from Dr. Bentong Yu (Department of Thoracic Surgery, the First Affiliated Hospital of Nanchang University). Samples were immediately stored in liquid nitrogen prior to experimental analysis. All samples were collected with patients‘ informed consent.

### Proteomic analysis

The central region and peripheral region of the tumors derived from lung adenocarcinoma patients were separated. Samples were ground into powder with liquid nitrogen and then the appropriate amount of RIPA buffer (Solarbio) containing PMSF (1 mM) is added. After mixed by vortexing, the lysates were broken by sonication. After centrifugation at 14000 *g* for 20 min, the supernatants were transferred to fresh tubes and stored at −80 °C. The supernatants were analyzed in Qlbio. Differentially expressed proteins were identifified through proteomic analysis.

### Cell culture

The NSCLC cell lines H1299 and A549 were obtained from ATCC and cultured in RPMI 1640 (Gibco) contained 10% fetal bovine serum (FBS) (ExCell Bio). For glucose starvation, cells were washed twice with PBS and further incubated in RPMI 1640 without glucose (Gibco) contained 10% FBS. All cells were cultured in 37 °C incubator with 5% CO_2_.

### q-PCR analysis

The q-PCR analysis assay was performed as previously described [54].

### Western blot and immunoprecipitation

The western blot and immunoprecipitation was performed as previously described [54].

### Flow cytometric analysis

Flow cytometric analysis of apoptotic cells was performed using the Annexin V-FITC / PI Apoptosis Detection Kit (Elabscience).

### Glycolysis stress test and Mito stress test

ECAR and OCR measurements were conducted using a Agilent Seahorse XFe24 analyzer according to manufacturer’s protocol. 1.5 × 10^4^ cells were seeded in XF24 cell culture microplate. For ECAR measurements, 10 mM glucose, 1.5 μM oligomycin, and 50 mM 2-deoxyglucose (2-DG) were injected at the indicated time points. For OCR measurements, 1.5 μM oligomycin, 2 μM FCCP and 0.5 μM rotenone/antimycin A were injected at the indicated time points.

### Measurement of lactic acid levels

Lactic acid levels were monitored using the lactic acid assay kit (Nanjing Jiancheng) according to the manufacturer’s protocol.

### Extraction of nuclear and cytoplasmic proteins

A Nuclear and Cytoplasmic Extraction Kit (Beyotime) was used to extract the nuclear and cytoplasmic proteins according to the manufacturer’s protocol.

### Mitochondria isolation

Mitochondria isolation was done using the mitochondria isolation kit (QIAGEN) according to the manufacturer’s protocol.

### MTT cell proliferation and cytotoxicity assay

5000 cells were cultured in 96-well plates in RPMI 1640 (200 µl) supplemented with 10% FBS. Then, cells were treated by glucose starvation for relevant interval. Next, 20 µl MTT (5 mg/ml) solution was added to cells and then incubated with 5% CO_2_ at 37 °C for 4 h. Then, the culture medium was replaced with 150 µl DMSO to incubated for 20 min. Finally, the absorbance was measured at 570 nm. Measurements were performed in triplicate.

### In vivo xenograft assay

1 × 10^7^ cells (100 μl suspensions) were injected subcutaneously into the flanks of 4-week-old male BALB/C nude mice (GemPharmatech). After 1 month, the mice were killed. Tumors were taken out and weighed and the volumes were measured according to the formula: volume (mm3) = (large diameter) × (smaller diameter)^2^ × π/6. All mice were housed in the SPF animal facility of the Institute of Life Science at Nanchang University and their care was in accordance with Nanchang University Animal Care Commission guidelines.

### Immunohistochemistry

Immunohistochemical staining of tumors derived from xenograft model was done as previously described [55]. Micrographs were taken by Olympus IX71 microscope.

### Statistical analysis

Data are shown as means ± SD. paired *t*-tests and One-way ANOVA were used for statistical comparisons. *P* < 0.05 was considered to be statistically significant.

## Supplementary information

Supplemental Figure legends

Supplementary Table 1

Supplementary Table 2

Supplementary Figure 1

Supplementary Figure 2

Supplementary Figure 3

Supplementary Figure 4

Supplementary Figure 5

Reproducibility checklist

## Data Availability

All datasets used and analyzed in the current study are available on reasonable request from the corresponding author.
