# Supplementary material for: 4EBP1 senses extracellular glucose deprivation and initiates cell death signaling in lung cancer
Source: Cell Death Dis. 2022 Dec 27;13(12):1075. doi: 10.1038/s41419-022-05466-5 (PMC9794714; doi:10.1038/s41419-022-05466-5)
Supplement: Supplementary file 1 — Supplemental Figure legends [file 41419_2022_5466_MOESM1_ESM.docx]

**Supplemental Figure legends**

**Fig. S1 Glucose deprivation induces dephosphorylation and stabilization of 4EBP1 in NSCLC cells.** **A** q-PCR analysis of 4EBP1 expression in A549 and H1299 cells under normal (0 h) or glucose starvation conditions for 3, 6, 9, 12 h. the data represent the averages of three independent experiments (mean ± SD). **B** A549 and H1299 cells transfected with plasmid His-K63-UB were treated with normal or glucose starvation (9 h). The immunoprecipitates using anti-4EBP1 antibody were blotted with the K63-linkage-specific polyubiquitin antibody. WCL: the whole cell lysate.

**Fig. S2 PTPMT1 dephosphorylates and stabilizes 4EBP1 under glucose deprivation conditions.** **A** Western blot analysis of PTPMT1 expression using the paired, tumor-central region (A) and tumor-peripheral region (B) derived from four lung adenocarcinoma patients. **B** Western blot analysis of PTPMT1 expression in H1299 and A549 cells under normal (0 h) or glucose starvation conditions for 3, 6, 9 and 12 h. **C** A549 cells transfected with either control (CTL) siRNA or PTPMT1 siRNAs were treated with CHX at 50 μg/ml for 0, 3, 6, 9, 12 and 24 h. The 4EBP1 expression was checked by western blot.

**Fig. S3 Glucose deprivation suppresses HERC5-mediated ubiquitination and degradation of 4EBP1. A** Western blot analysis of HERC5 expression in H1299 and A549 cells under normal (0 h) or glucose starvation conditions for 3, 6, 9 and 12 h. **B** Western blot analysis of HERC5 expression using the paired, tumor-central region (A) and tumor-peripheral region (B) derived from four lung adenocarcinoma patients. **C, D** A549 cells were transfected with plasmid HA-HERC5 or vector. The 4EBP1 expression was checked by western blot (**C**) or q-PCR (**D**).

**Fig. S4 4EBP1 plays a crucial role in cell apoptosis under glucose deprivation conditions.** **A** Western blot analysis of p-eIF4E-S209 expression in H1299 and A549 cells under normal (0 h) or glucose starvation conditions for 3, 6, 9 and 12 h. **B** Western blot analysis of p-eIF4E-S209 expression using the paired, tumor-central region (A) and tumor-peripheral region (B) derived from four lung adenocarcinoma patients. **C, D** H1299 cells transfected with either control (CTL) siRNA or 4EBP1 siRNAs were treated with normal or glucose starvation (20 h). (**C**) The changes of cell morphological was investigated (scale bar = 100 µm, magnification: 100×). (**D**) Cell apoptosis analysis was done by flow cytometry.

**Fig. S5 4EBP1 inhibits tumor progression under glucose deprivation conditions in vivo. A** Immunohistochemical staining in the central region and peripheral region in xenografts of parental A549 cells for 4EBP1, p-STAT3, BCL2, MCL1, Survivin and Cleaved PARP1 (C-PARP1). Scale bars: 50 μm, magnification: 200×.
