# Supplementary figures and images for: 4EBP1 senses extracellular glucose deprivation and initiates cell death signaling in lung cancer
Source: Cell Death Dis. 2022 Dec 27;13(12):1075. doi: 10.1038/s41419-022-05466-5 (PMC9794714; doi:10.1038/s41419-022-05466-5)

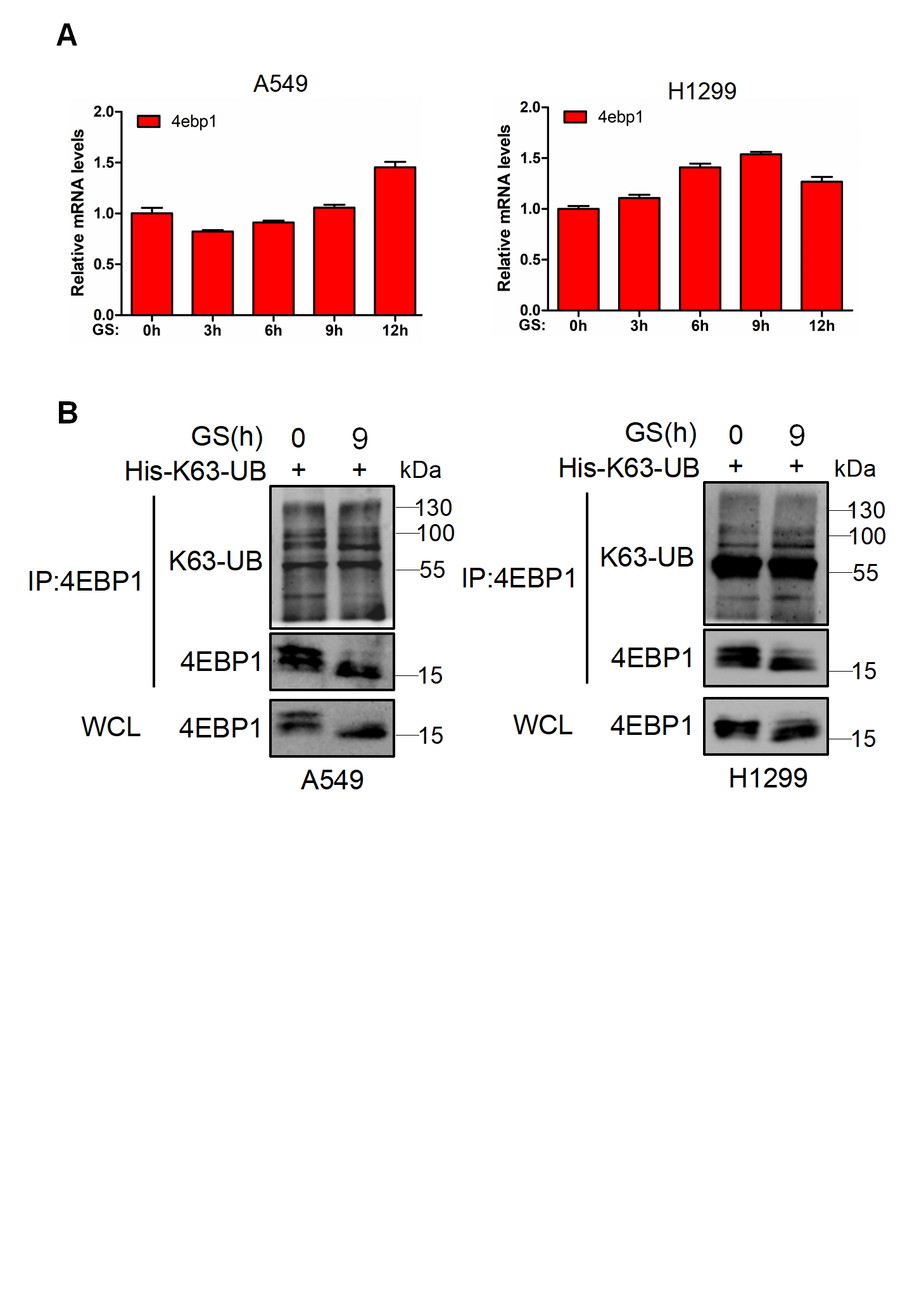

Supplement: Supplementary file 4 — Supplementary Figure 1 [file 41419_2022_5466_MOESM4_ESM.tif]

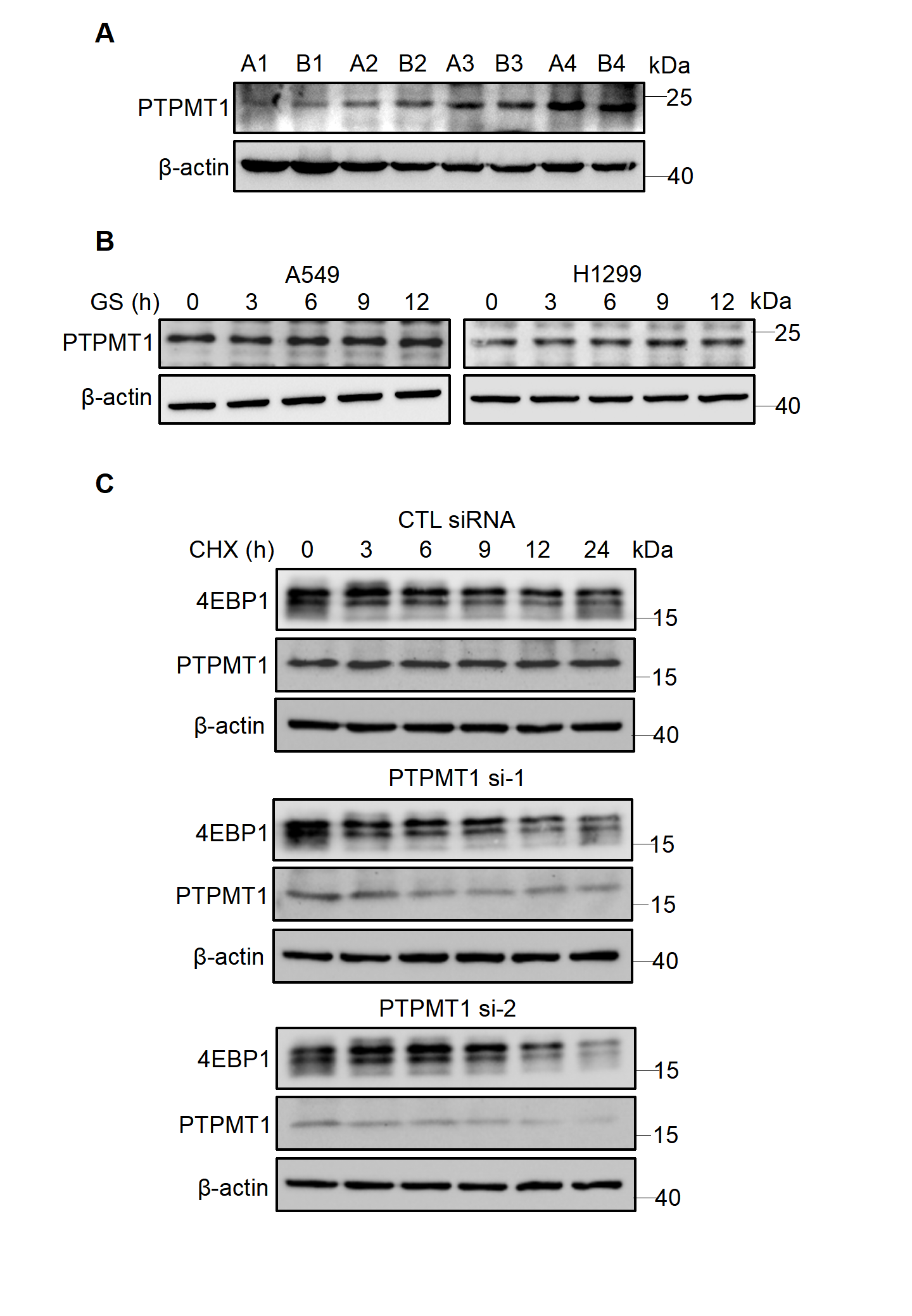

Supplement: Supplementary file 5 — Supplementary Figure 2 [file 41419_2022_5466_MOESM5_ESM.tif]

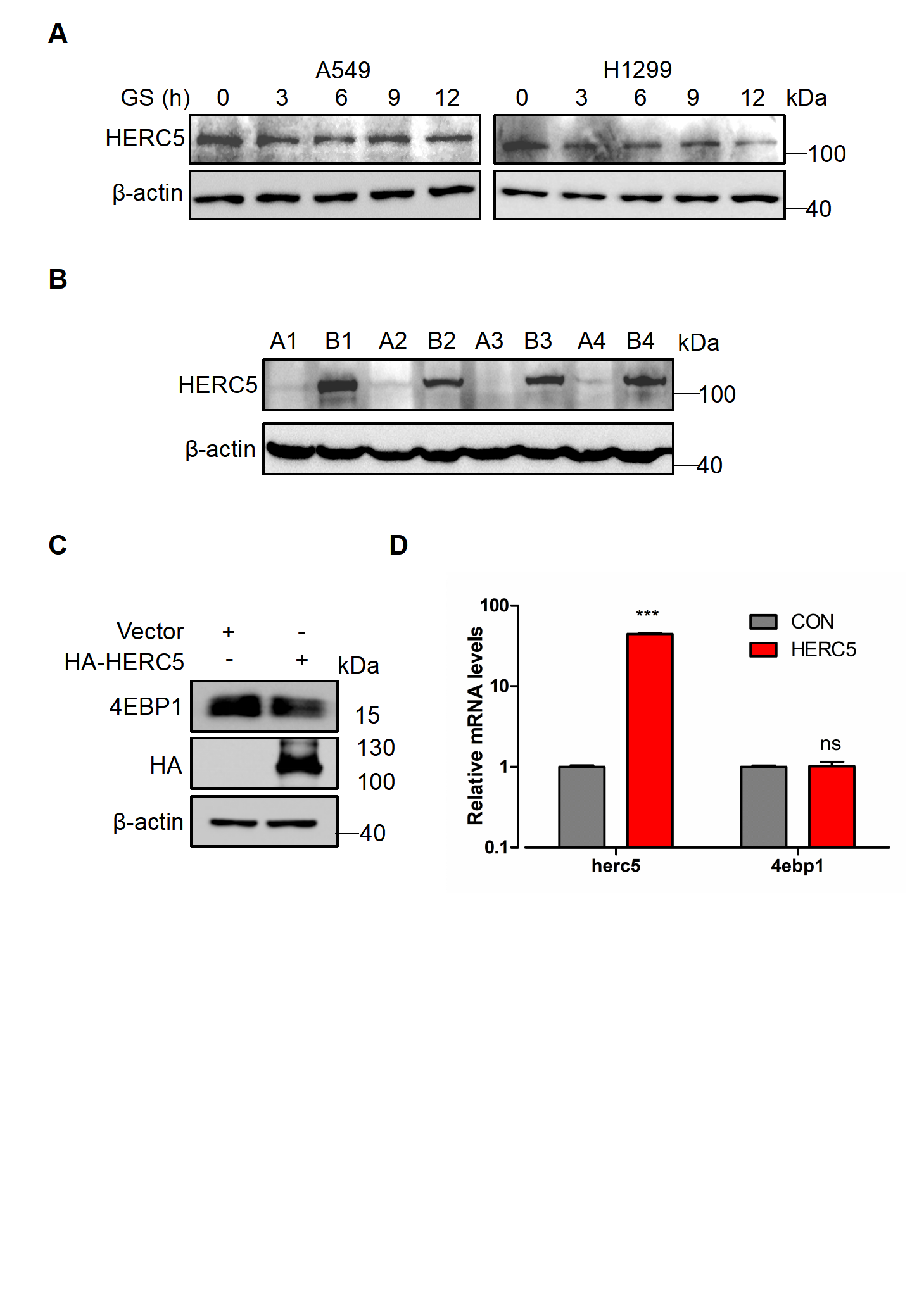

Supplement: Supplementary file 6 — Supplementary Figure 3 [file 41419_2022_5466_MOESM6_ESM.tif]

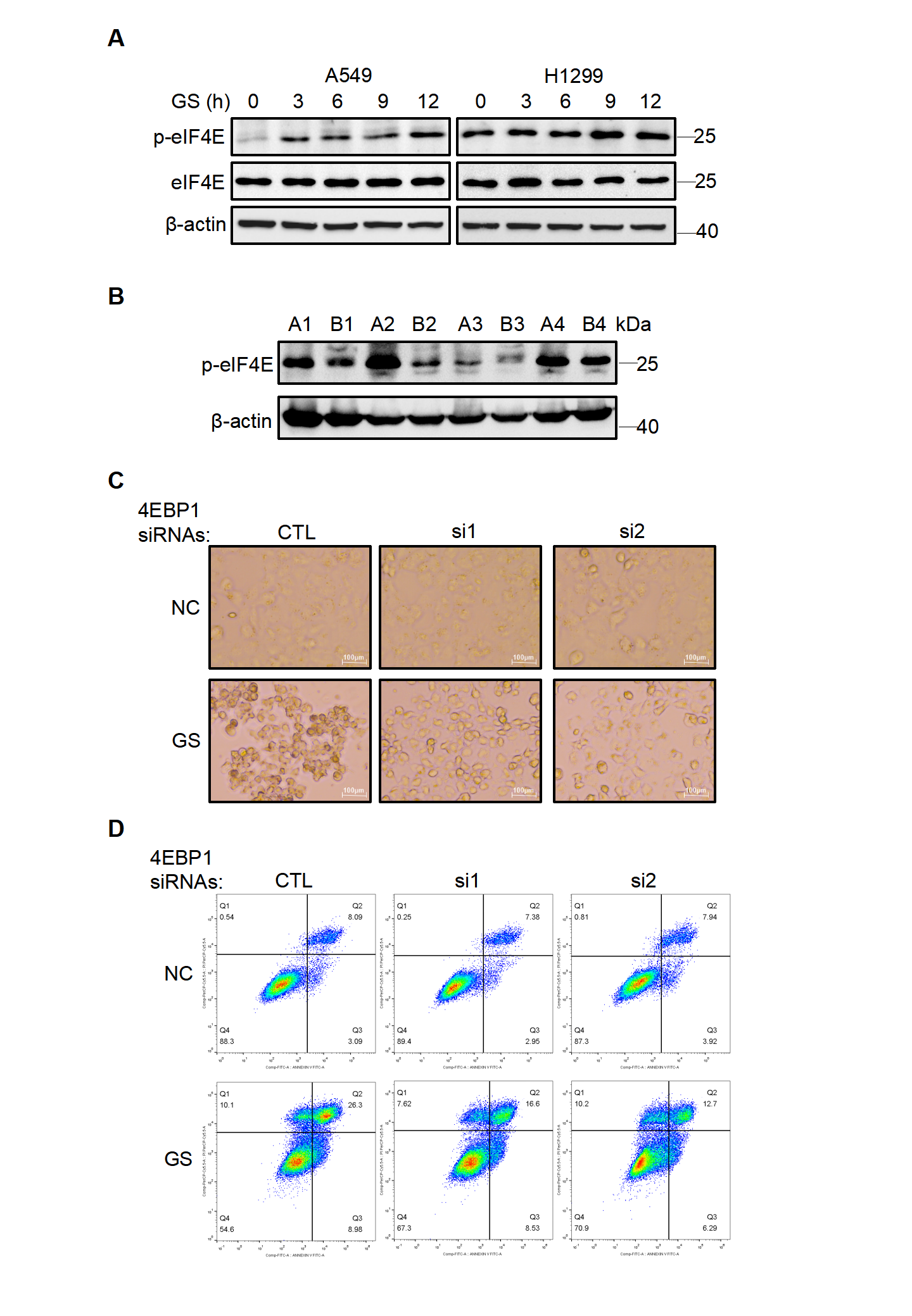

Supplement: Supplementary file 7 — Supplementary Figure 4 [file 41419_2022_5466_MOESM7_ESM.tif]

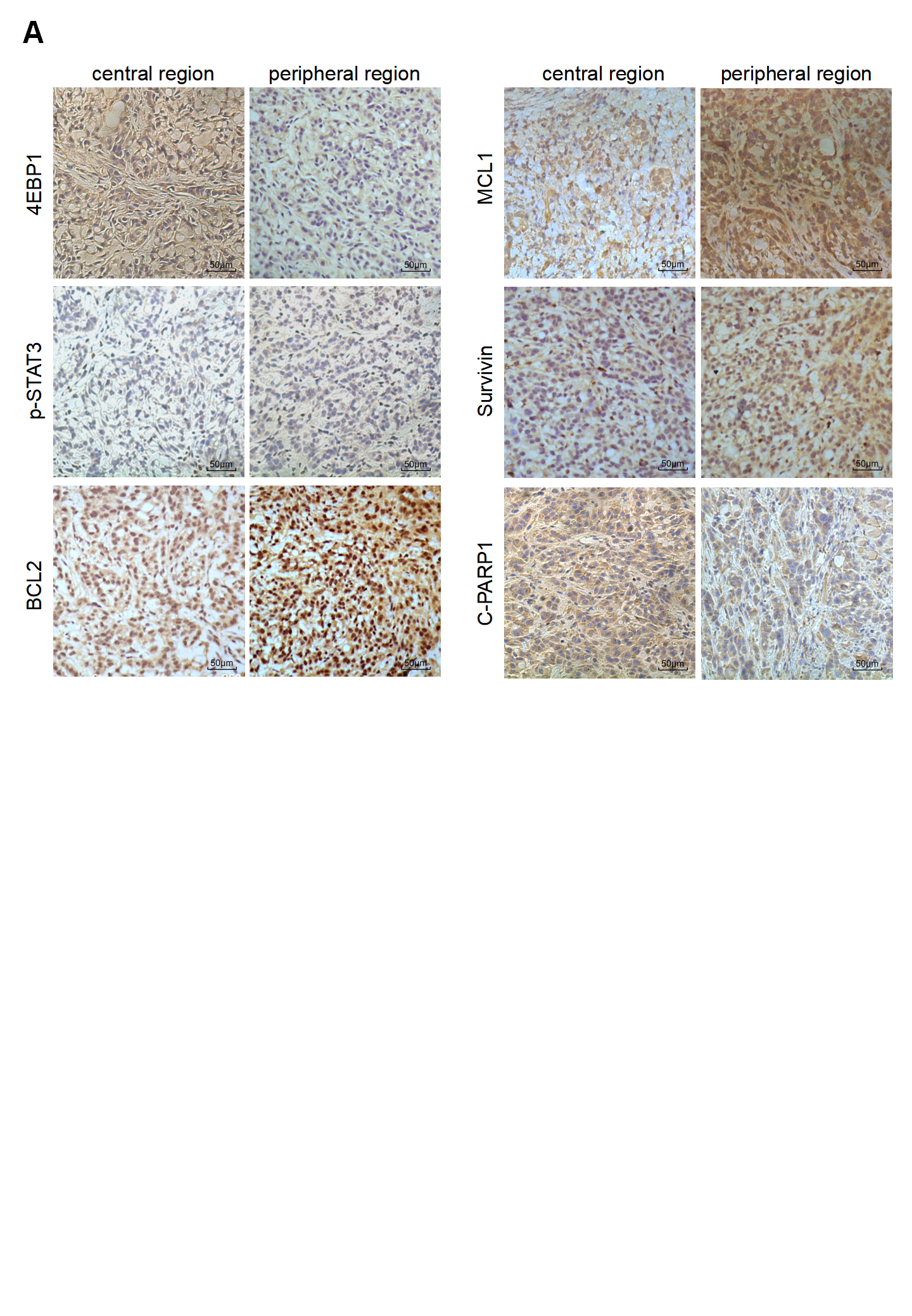

Supplement: Supplementary file 8 — Supplementary Figure 5 [file 41419_2022_5466_MOESM8_ESM.tif]
